# The complete chloroplast genome of *Styrax confusus* Hemsl. (Styracaceae)

**DOI:** 10.1080/23802359.2020.1715890

**Published:** 2020-01-24

**Authors:** Xiaogang Xu, Yaoqin Zhang, Lili Tong, Yabo Wang, Chongli Xia

**Affiliations:** a Co-Innovation Center for Sustainable Forestry in Southern China, College of Biology and the Environment, Key Laboratory of State Forestry and Grassland Administration on Subtropical Forest Biodiversity Conservation, Nanjing Forestry University, Nanjing, China;; b State Environmental Protection Scientific Observation and Research Station for Ecology and Environment of Wuyi Mountains, Nanping, China; c School of Horticulture and Landscape Architecture, Jinling Institute of Technology, Nanjing, China

**Keywords:** *Styrax confusus*, Styracaceae, complete chloroplast genome, phylogenomics

## Abstract

*Styrax confusus* Hemsl. is valued for its beauty and fragrance. Here, we characterized the complete chloroplast (cp) genome of *S*. *confusus* using next-generation sequencing. The circular complete cp genome of *S. confusus* was 157,981 bp in length, containing a large single-copy (LSC) region of 87,571 bp, and a small single-copy (SSC) region of 18,316 bp. It comprises 133 genes, including eight rRNA genes, 37 tRNA genes, and 88 protein-coding genes. The GC content of *S. confusus* cp genome is 36.95%. A phylogenetic tree reconstructed by 30 chloroplast genomes reveals that *S. confusus* is mostly related to *Styrax calvescens* Perk.

*Styrax confusus* Hemsl., a shrub or small tree with abundant white fragrant flowers blooming in late spring, is a multipurpose economic arbor species integrating ornamental, oil and biomass energy values. Here, we characterized the complete cp genome sequence of *S. confusus* (GeneBank accession number: MN560142) based on Illumina pair-end sequencing to provide a valuable complete cp genomic resource.

Total genomic DNA was isolated from fresh leaves of *S. confusus* grown in Shimenxia (N 30.8449, E 118.1420, Alt. 322 m), Huangshan, in Anhui, China. The voucher specimen was deposited at the herbarium of Nanjing Forestry University (accession number NF2019903). Illumina paired-end (PE) DNA library was prepared and sequenced in Nanjing Genepioneer Biotechnologies Inc., Nanjing, China. Raw reads were trimmed using CLC Genomics Workbench v9 and the resultant clean reads were then employed to assemble the cp genome using the program SPAdes assembler v3.10.1 (Bankevich et al. 2012). The resultant genome was annotated by CpGAVAS (Liu et al. 2012). The sequences were aligned by MAFFT v7.307 (Katoh and Standley 2014). A Neighbor-Joining (NJ) tree with 100 bootstrap replicates was inferred using MEGA version 7 (Kumar et al. 2016).

The circular genome of *S. confusus* was 157,981 bp in size and contained two inverted repeat (IRa and IRb) regions of 26,047 bp, which were separated by a large single copy (LSC) region of 87,571 bp, and a small single copy (SSC) region of 18,316 bp. A total of 133 genes are encoded, including 88 protein-coding genes (81 PCG species), 37 tRNA genes (30 tRNA species), and eight rRNA genes (4 rRNA species). Most of the genes occurred in a single copy, however, 7 protein-coding genes (*ndhB, rpl2, rpl23, rps12, rps7, ycf15,* and *ycf2*), 7 tRNA genes (*trnA-UGC, trnI-CAU, trnI-GAU, trnL-CAA, trnN-GUU, trnR-ACG,* and *trnV-GAC)* , and 4 rRNA genes (*4.5S, 5S, 16S,* and *23S*) are totally duplicated. A total of 9 protein-coding genes (*atpF, ndhA, ndhB, petB, petD, rpl16, rpl2, rpoC1,* and *rps16*) contained one intron while the other 3 genes (*clpP, rps12, and ycf3*) had 2 intron each. The overall GC content of *S. confusus* genome is 36.95% and the corresponding values in LSC, SSC, and IR regions are 34.80, 30.22, and 42.93%, respectively.

In order to reveal the phylogenetic position of *S. confusus* with other members of Styracaceae, a phylogenetic analysis was performed based on 25 complete chloroplast genomes of Styracaceae, and 3 taxa (Symplocaceae, Clethraceae, and Ericaceae) as outgroups. They were all downloaded from NCBI GenBank. We found that *S. confusus* was clustered with *Styrax calvescens* Perk. with 80% bootstrap values (Figure 1).

**Figure 1.**
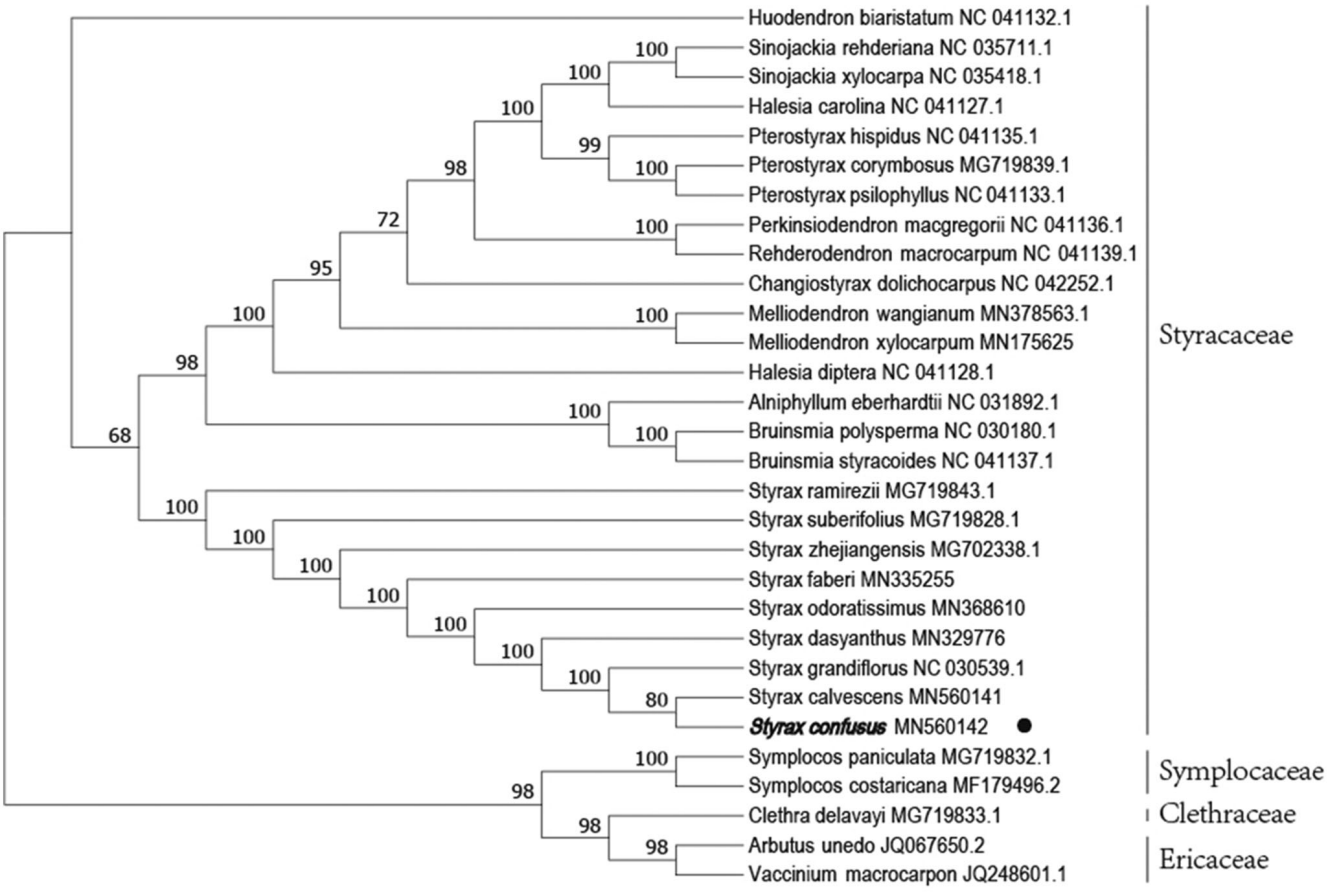
Phylogenetic tree inferred by neighbor-joining (NJ) method based on the complete chloroplast genome of 30 representative species. The bootstrap support values are shown at the branches.
